# Methionine deficiency causes spermatogonial apoptosis via oxidative stress and DNA damage response pathway

**DOI:** 10.1186/s40659-025-00652-z

**Published:** 2025-11-12

**Authors:** Weiyong Wang, Yong Ruan, Gong Ting

**Affiliations:** Key Laboratory of Animal Genetics, Breeding and Reproduction in the Plateau Mountainous Region, Ministry of Education, Guizhou University, Guiyang, 550025 Guizhou China

**Keywords:** Methionine, Spermatogenesis, Apoptosis, DNA damage, Oxidative stress

## Abstract

Methionine serves as an essential amino acid regulating de novo protein synthesis and redox homeostasis. Previous studies have established adverse impacts of methionine restriction and deprivation on semen quality, but effects on early spermatogenesis remain poorly characterized. In this study, a methionine dietary model (0.86%, 0.17%, 0%) was used to investigate the role of methionine in early spermatogenesis. The results indicated that methionine deprivation caused spermatogenesis defects by inhibiting spermatogonial proliferation and increasing apoptosis. Further studies showed that methionine deprivation downregulated mitochondrial function-related genes (*Gpx4*, *Fis1* and *Gstm1*), but upregulated ISR- (*Atf4*, *Chac1* and *Ddit3*) and DNA damage response-related genes (*Cdkn1a*, *Chek2* and *Atm*). Meanwhile, methionine deprivation caused mitochondrial dysfunction characterized by mitochondrial membrane potential depolarization, ROS accumulation, and MitoSOX accumulation. Methionine deprivation also caused an obvious increase in DNA damage response proteins (γH2AX, p-CHK2 and p-p53) and pro-apoptotic proteins (PUMA, BAX and c-PARP1), but suppressed anti-apoptotic protein BCL2. Furthermore, NAC effectively reversed the proliferation deficiency of GC-1 cells caused by methionine deprivation. Collectively, these findings suggest that methionine deprivation triggers ISR activation, which subsequently induces spermatogonial apoptosis via oxidative stress and the CHK2-p53/p21 signaling cascade. This study highlights the critical role of methionine in early spermatogenesis, provides mechanistic insights for optimizing dietary interventions and addresses related reproductive disorders.

**Supplementary Information:**

The online version contains supplementary material available at 10.1186/s40659-025-00652-z.

## Introduction

Methionine is an essential amino acid, indispensable for vital cellular processes such as protein and DNA synthesis, antioxidant processes, and epigenetic modifications [1]. Methionine ingested from food is broken down and metabolized by the methionine cycle to maintain amino acid homeostasis in vivo [2]. Unbalanced methionine intake and insufficient absorption and metabolism can cause metabolic diseases, such as hypermethioninemia and methionine malabsorption syndrome [3, 4]. Furthermore, methionine synthesis defects caused by vitamin B12 deficiency can cause hyperhomocysteinemia, megaloblastic anemia, Alzheimer´s disease and involuntary movement [5]. In recent years, many studies have found that dietary methionine restriction plays a beneficial role in cancer treatment, lifespan extension and fertility improvement by reducing oxidative stress, inhibiting protein synthesis and inducing unique metabolic protection [6, 7]. However, the mechanisms are very complex and not yet fully understood.

In fact, as a common response pathway, amino acid deprivation could activate the downstream general control nonderepressible 2 (GCN2), which further phosphorylates eukaryotic translational initiation factor 2α (eIF2α) at Ser 51 [8]. The increased phosphorylation of eIF2α drives translation reprogramming, characterized by inhibition in global translation and increase in the selective translation of mRNA critical for cell survival and apoptosis, such as activating transcription factor 4 (ATF4), DNA-damage inducible transcript 3 (DDIT3) and fibroblast growth factor 21 (FGF21), to help cells to restore protein homeostasis under stress [9, 10]. In addition, amino acid deprivation can inhibit the mTOR pathway and further initiate autophagy and activate the integrated stress response to maintain cell survival. However, the methionine deprivation in QM7 cells and HEK293T cells causes a drastic decrease in translation but is independent of the GCN2-eIF2α signaling cascade [11, 12]. Further studies showed that the decrease in protein synthesis and activation in ATF4 and its target genes could be attributed to inhibition of mTOR-S6K1 signaling [12, 13]. In cancer cells, short-term rather than long-term methionine starvation promotes the transcription of cation transport regulator homolog 1 (CHAC1) to induce ferroptosis, which depends on glutathione depletion [14]. Further in-depth studies propose that methionine restriction or deprivation could activate the PKR-like endoplasmic reticulum kinase (PERK) and downstream eIF2α-NRF2 signal transduction via GSH-sensitive mechanism [15, 16]. Altogether, these studies above indicate that methionine restriction or deprivation results in multiple phenotypes by different molecular mechanisms.

The testis, as an indispensable reproductive organ in males, plays an important role in steroidogenesis and spermatogenesis [17]. The former mainly occurs in the Leydig cells, while the latter is mainly controlled by the orderly development of different stages of germ cells in the seminiferous epithelium [18, 19]. Spermatogenesis failure is associated with functional defects in Leydig cells, germ cells, or both, which are caused by genetic mutations, environmental pollution and an unbalanced diet [20]. Previous studies showed that methyl donor S-adenosyl-methionine (SAM) can increase the LH/hCG binding sites on rat Leydig cells to promote the production of cAMP and testosterone in vitro [21]. However, excessive methionine intake causes a decrease in the enzymatic activity of 3β-Hydroxysteroid Dehydrogenase and a loss of germ cells [22, 23], but the mice receiving a methionine-choline-deficient diet show normal testosterone synthesis ability and a complete blood-testis barrier [24]. Interestingly, Jason W. Locasale and his colleague have reported that lowering dietary methionine levels can reduce fertility, and in particular, a diet completely deprived of methionine results in significantly fewer offspring [25]. Further studies showed that methionine restriction or deprivation decreased the sperm count and adversely affected sperm motility [25]. These studies suggest that methionine intake is critical for male fertility maintenance. However, systematic research is still lacking on the effects of methionine restriction and deprivation during early spermatogenesis. Moreover, the specific mechanisms responsible for male fertility defects caused by insufficient or excessive methionine intake also need to be elucidated.

The effects of nutrition on animal production and human health and longevity will continue to be one of the most important issues facing society in the future. Amino acid dietary restrictions (such as methionine) showed active contribution to disease prevention and intervention as well as life-span extension, but the negative effects on weight, bone development, reproduction and other health issues cannot be ignored [26–28]. Here, the in vivo dietary manipulation (methionine restriction/deprivation) in immature mice and in vitro cell experiments were used to explore the contribution of methionine to early testicular development.

##  Materials and methods

### Animals

All C57BL/6J male mice at postnatal days (PD)25 were purchased from Guizhou Medical Laboratory Animal Center and then housed in an individual ventilated cage with a suitable temperature (22 ± 1 °C), humidity (50–70%), and a 12/12 h light/dark cycle. To achieve methionine restriction or deprivation in mice, we designed and produced three types of model feed with different methionine contents: 0.86% (normal control group, Met^+^), 0.17% (methionine restriction, MR), and 0% (methionine deprivation, Met^−^). The detailed feed formula and the nutritional levels were shown in Supplementary Table 1. It is notable that the diets were isocaloric and sulfur amino acid compensation was ensured. Subsequently, 18 25-day-old male mice (9 ~ 12 g) were randomly divided into three groups using the blind method and fed with the model feed for 14 days, each group containing 6 mice. After this, all mice at PD40 were anesthetized by intraperitoneal injection of 400 mg/kg of tribromoethanol (MA0478, Meilunbio, China), and then euthanasia was performed by cervical dislocation. Finally, the testes were collected via a surgical procedure by an independent researcher who was unaware of the experimental group. All animal protocols were approved by the Institutional Animal Care and Use Committee of Guizhou University.

**Table 1:** Methionine model feed formulation

L-AA Defined AlN-93G	Methionine: 0.86%		Methionine: 0.17%		Methionine: 0%	
Ingredient	g/kg	kcal	g/kg	kcal	g/kg	kcal
L-Arginine	6.3	25.2	6.3	25.2	6.3	25.2
L-Histidine	4.5	18	4.5	18	4.5	18
L-Lysine HCl	16.1	64.4	16.1	64.4	16.1	64.4
L-Tyrosine	9.2	36.8	9.2	36.8	9.2	36.8
L-Tryptophan	2.1	8.4	2.1	8.4	2.1	8.4
L-Phenylalanine	8.7	34.8	8.7	34.8	8.7	34.8
**L-Methionine**	**8.6**	**34.4**	**1.7**	**6.8**	**0**	**0**
L-Cystine	3.7	14.8	3.7	14.8	3.7	14.8
L-Threonine	6.6	26.4	6.6	26.4	6.6	26.4
L-Leucine	15.3	61.2	15.3	61.2	15.3	61.2
L-lsoleucine	8.4	33.6	8.4	33.6	8.4	33.6
L-Valine	9.9	39.6	9.9	39.6	9.9	39.6
Glycine	3.1	12.4	3.1	12.4	3.1	12.4
L-Proline	20.4	81.6	20.4	81.6	20.4	81.6
L-Glutamic Acid	36.2	144.8	36.2	144.8	36.2	144.8
L-Alanine	4.5	18	4.5	18	4.5	18
L-Aspartic Acid	17.3	45.2	17.3	45.2	17.3	45.2
L-Serine	9.4	37.6	9.4	37.6	9.4	37.6
**Total L-Amino Acid**	**184.3**	**737.2**	**184.3**	**709.6**	**184.3**	**702.8**
Cornstarch	395.8	1583.2	402.7	1610.8	404.4	1617.6
Maltodextrin10	145	580	145	580	145	580
Sucrose	100	400	100	400	100	400
Cellulose	50	0	50	0	50	0
Soybean Oil	70	630	70	630	70	630
Choline Chloride	2.5	0	2.5	0	2.5	0
Mineral Mix S10022G	35	0	35	0	35	0
Vitamin Mix V10037	10	40	10	40	10	40
Sodium Bicarbonate	7.4	0	7.4	0	7.4	0
FD&C Red Dye #40	0	0	0.05	0	0	0
FD&C Yellow Dye #5	0	0	0	0	0.05	0
FD&C Blue Dye #1	0	0	0	0	0	0
**Total**	**1000**	**3970.4**	**1000**	**3970.4**	**1000**	**3970.4**

### Histomorphological analysis

The testes and epididymides of mice were surgically separated and fixed in 4% paraformaldehyde for 24 h at 4 °C. Subsequently, the testes and epididymides were dehydrated in graded alcohols, cleared in xylene, embedded in paraffin, and sectioned into 5-µm-thick slices. These tissue sections were stained with Hematoxylin and Eosin, and then the images were captured using an upright microscope.

### Immunofluorescence staining

Testicular sections underwent dewaxing and rehydration in xylene and graded alcohols, respectively. Next, these sections were immersed in sodium citrate buffer (pH = 6.0) for antigen retrieval at high temperatures. To block non-specific binding, the sections were exposed to 5% bovine serum albumin (BSA) for 1 h at room temperature, then incubated with primary antibodies at 4℃ overnight. After three washes with PBS, these sections were treated with secondary antibodies conjugated with Alexa Fluor 488, 594, or 647 at 37℃ for 1 h. Finally, the nucleus was counterstained by 4,6-diamidino-2-phenylindole (DAPI), and the images were captured with a confocal microscope.

### In vivo BTB integrity analysis

The in vivo BTB integrity analysis was performed as previously described [29]. Briefly, the anesthetized mice received an intratesticular injection of 20 µL of biotin (10 mg/mL) dissolved in phosphate-buffered solution containing 1 mM CaCl_2_. After 30 min, the testes were excised and underwent fixation, sucrose sedimentation, and optimal cutting temperature (OCT)-embedding to prepare 5 μm-thick sections using a cryostat. Finally, these sections were fixed using pre-cooled − 80 °C methanol for 30 min and followed by incubation with FITC-labeled streptavidin solution for 1 h at room temperature. The cell nuclei were visualized with DAPI.

### TUNEL staining

Testicular cell apoptosis was detected using TUNEL staining. Specifically, the dewaxed and rehydrated sections were permeabilized with proteinase K for 30 min. After three washes, the sections were incubated with a TUNEL reaction mixture for 1 h at 37℃ in the dark. Finally, the nucleus was counterstained by DAPI, and the images were captured with a confocal microscope.

### Cell culture and treatment

The GC-1 cells were cultured and maintained in the DMEM medium supplemented with 10% fetal bovine serum (FBS) and 100 IU penicillin and streptomycin. After the cells adhered to the wall, the methionine-deprived groups were cultured in the methionine-free DMEM (C0891, Beyotime) supplemented with glutamine and 10% dialyzed serum. In contrast, the cell culture medium of the control groups was supplemented with glutamine, L-methionine and 10% dialyzed serum.

### RNA sequencing

The GC-1 cells underwent methionine deprivation and the mRNA libraries were submitted to the Illumina HiSeq 4000 platform for sequencing. The raw data contains reads of low quality, reads with adaptor sequences and reads with high levels of N base were filtered by Cutadapt (v1.9) to obtain clean reads, and then aligned with the reference genes GRCm39 using HISAT2 (v2.0.4). Finally, the FPKM (fragments per kilobase of exon model per million mapped fragments) was calculated and standardized. Gene expression differences were evaluated using the DESeq2 package in R, with candidate genes defined as those exhibiting a q-value ≤ 0.05 and a log2 fold change ≥ 1.

### RNA extraction and RT-qPCR

Total RNA was extracted from the tests using Trizol reagent, and the RNA concentration and purity were detected using NanoDrop 2000. Subsequently, 2000 ng of RNA was used to generate the cDNA using a Reverse Transcription Kit, and the quantitative real-time polymerase chain reaction (RT-qPCR) was performed to quantify mRNA levels. The *Actb* (β-actin) was selected as an internal control to normalize the RT-qPCR data, and relative mRNA expression was calculated by the 2^−∆∆Ct^ method. All the primer sequences for RT-qPCR were listed in Table S1.

### Western blot analysis

Total protein was extracted from the testis using RIPA with PMSF and protease inhibitor cocktail. 20 µg of denatured protein samples were separated by the SurePAGE™ (10%, GenScript) and transferred to PVDF membranes. The membranes were blocked in TBST with 5%BSA at room temperature for 1 h before incubating with primary antibodies overnight at 4℃. After three washes with PBST, the membranes were treated with secondary antibodies for 1 h. Finally, the bands were visualized in the Tanon 5200 imaging system with ECL chromogenic solutions. The grayscale values of these bands were quantified using ImageJ software within the linear range of detection and then normalized by GAPDH and α-tubulin. All antibody information is available in Table S2.

### Mitochondrial membrane potential assay

The GC-1 cells were cultured in a 4-chamber glass-bottom dish and underwent methionine deprivation treatment. Then, the cells were incubated with 2 µmol/L JC-1 (MT09, DOJINDO) in DMEM medium for 30 min at 37℃, and subsequently washed using Hanks balanced salt solution (HBSS) twice. Finally, the Imaging Buffer Solution was added and submitted to an LSM 800 confocal microscope to capture images.

### Reactive oxygen species and mitochondrial superoxide detection

The reactive oxygen species (ROS) levels were detected using a ROS assay kit -highly sensitive DCFH-DA (R253, DOJINDO) according to the protocols. In brief, the cultured cells were treated with DCFH-DA in loading buffer solution at 37℃ for 30 min, then washed using HBSS twice and submitted to an LSM 800 confocal microscope to capture images. Mitochondrial superoxide (MitoSOX) was detected using the Mitochondrial Superoxide indicator (RM02822, Abclonal), and the detection procedure was carried out following the previously established procedures [30].

### Intracellular ferrous ion detection

The intracellular ferrous ion levels were detected using the FerroOrange indicator (F374, DOJINDO) according to the protocols. In brief, the cultured cells were firstly washed three times using HBSS and then treated with 2 µmol/L FerroOrange at 37℃ for 30 min. After these, the cells were directly submitted to an LSM 800 confocal microscope to capture images.

### Lipid peroxidation detection

The lipid peroxidation was detected using Lipid peroxidation probe-BDP 581/591 C11 (L267, DOJINDO) according to the protocols. In brief, the cultured cells were firstly washed twice using DMEM medium and then treated with BDP 581/591 C11 working solution at 37℃ for 30 min. Finally, the cells were washed twice using HBSS and submitted to an LSM 800 confocal microscope to capture images.

### Statistical analysis

All experiments were repeated at least three times and the experimental data were displayed as mean ± standard deviation (SD). A two-sided Student’s t‐test was used to determine *p*‐values for two groups, while one-way ANOVA followed by a Tukey’s post-hoc test was used for three or more groups. The data were visualized with GraphPad Prism. Differences were considered significant at *p* < 0.05 (*p < 0.05, **p < 0.01, ***p < 0.001, and ****p < 0.0001).

## Results

### Methionine deprivation but not methionine restriction blocks spermatogenesis in mice

**Fig. 1:**
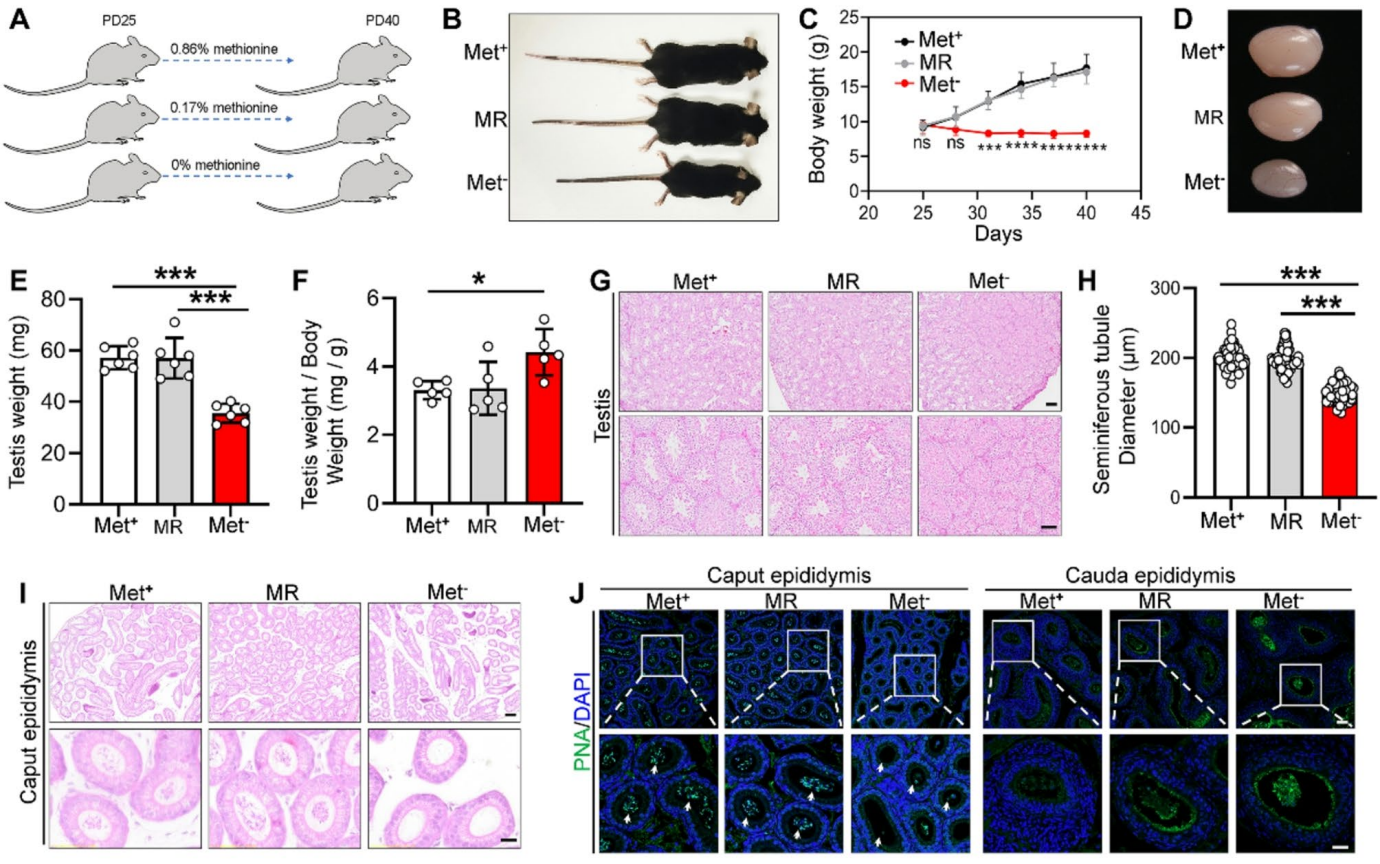
Methionine deprivation leads to spermatogenesis defects. **A** Schematic diagram of the methionine diet control model. **B** Representative images of mice undergoing a methionine diet control at PD40, *n* = 6. **C** Time-dependent changes in mouse body weight from PD25 to PD40, *n* = 6. **D** Representative images of the testes undergoing the methionine diet control at PD40. **E** Testis weight (*n* = 6) and index (*n* = 5) in Met^+^, MR and Met^−^ mice. **G** HE staining of testis from Met^+^, MR and Met^−^ mice. **H** The seminiferous tubule diameter in Met^+^ (68 seminiferous tubules from 3 mice), MR (78 seminiferous tubules from 3 mice) and Met^−^ (73 seminiferous tubules from 3 mice) mice. **I** HE staining of the caput epididymis from Met^+^, MR and Met^−^ mice. **J** Immunofluorescence staining of PNA in the caput epididymis and cauda epididymis from Met^+^, MR and Met^−^ mice. Cell nuclei were visualized using DAPI. Bars indicate the mean ± SD. A two-sided Student’s t‐test was used to determine *p*‐values for two groups, while one-way ANOVA followed by a Tukey’s post-hoc test was used for three or more groups. (*p < 0.05 and ***p < 0.001). Scale bar = 100 μm

To evaluate the effects of the methionine dietary restrictions on early testicular development, we subjected mice to methionine restriction and deprivation (Fig. 1A). The results showed that the body size and weight, testicular size and weight of the mice in the methionine restriction group were not significantly different from those in the control group (Fig. 1B-F and Table S3). However, the body size and weight, testicular size and weight of the mice in the methionine deprivation group showed a significant reduction compared with those of the mice in the control group (Fig. 1B-F and Table S3). Further, HE staining revealed that there was no significant difference in morphology between control and methionine restriction groups, while the testis in the methionine deprivation group showed histopathological alterations and spermatogenesis defects, including decreased diameter in seminiferous tubules, large-area closed seminiferous tubules and delayed spermatogenesis (Fig. 1G and H). Consistent with these findings, HE and PNA staining both showed that a certain number of sperm were observed in the caput epididymis of mice in the control and methionine restriction groups, but almost none were observed in the caput epididymis of mice in the methionine deprivation group (Fig. 1I and J). Furthermore, no sperm were observed in the cauda epididymis of all mice in all groups (Fig. 1J and Figure S1), probably because the mice had just reached puberty [31, 32].

### Methionine deprivation downregulates the expression of partial steroidogenic enzymes

**Fig. 2:**
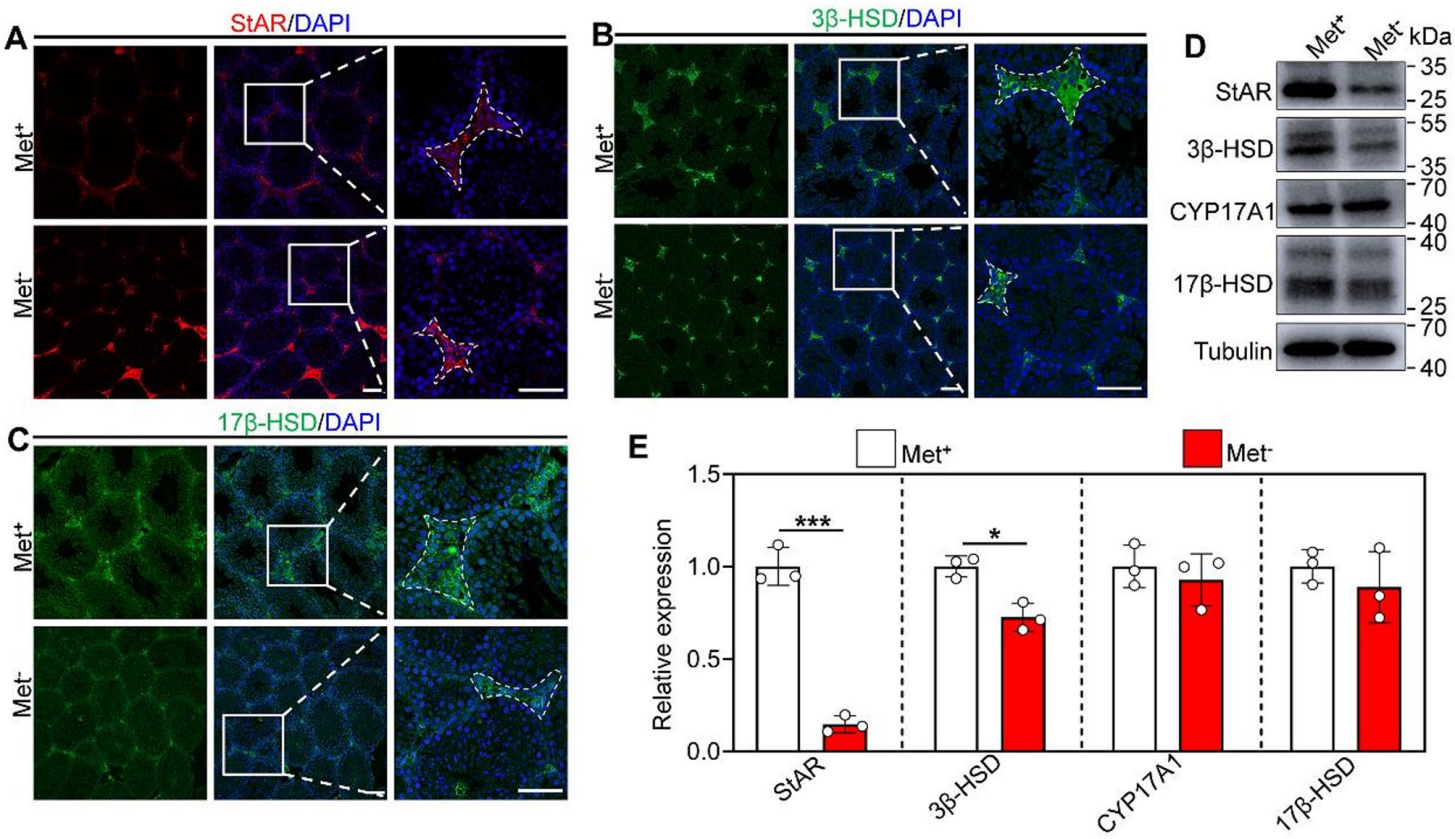
Methionine deprivation impairs steroidogenesis. **A**, **B**, **C** Immunofluorescence staining of StAR, 3β-HSD and 17β-HSD in testis from Met^+^, MR and Met^−^ mice. Cell nuclei were visualized using DAPI. (D, E) Western blot analysis of StAR, 3β-HSD, 17β-HSD and CYP17A1 protein levels in testis from Met^+^ and Met^−^ mice, *n* = 3. Bars indicate the mean ± SD. A two-sided Student’s t‐test was used to determine *p*‐values. (*p < 0.05 and ***p < 0.001). Scale bar = 100 μm

Given the previously reported differential effects of methionine deprivation on Leydig cells, we performed immunofluorescence staining and Western blot analysis on the steroidogenic enzymes. The results showed that the protein levels of Steroidogenic acute regulatory protein (StAR) and 3 beta-hydroxysteroid dehydrogenases (3β-HSD) were significantly decreased in the mouse testis in the methionine deprivation group compared with the control group (Fig. 2A-E). However, the 17β-HSD and cytochrome P450, family 17, subfamily a, polypeptide 1(CYP17A1) did not show differential expression in the mouse testes with methionine deprivation compared with the control group (Fig. 2A-E).

### Methionine deprivation does not affect the function of Sertoli cells

**Fig. 3:**
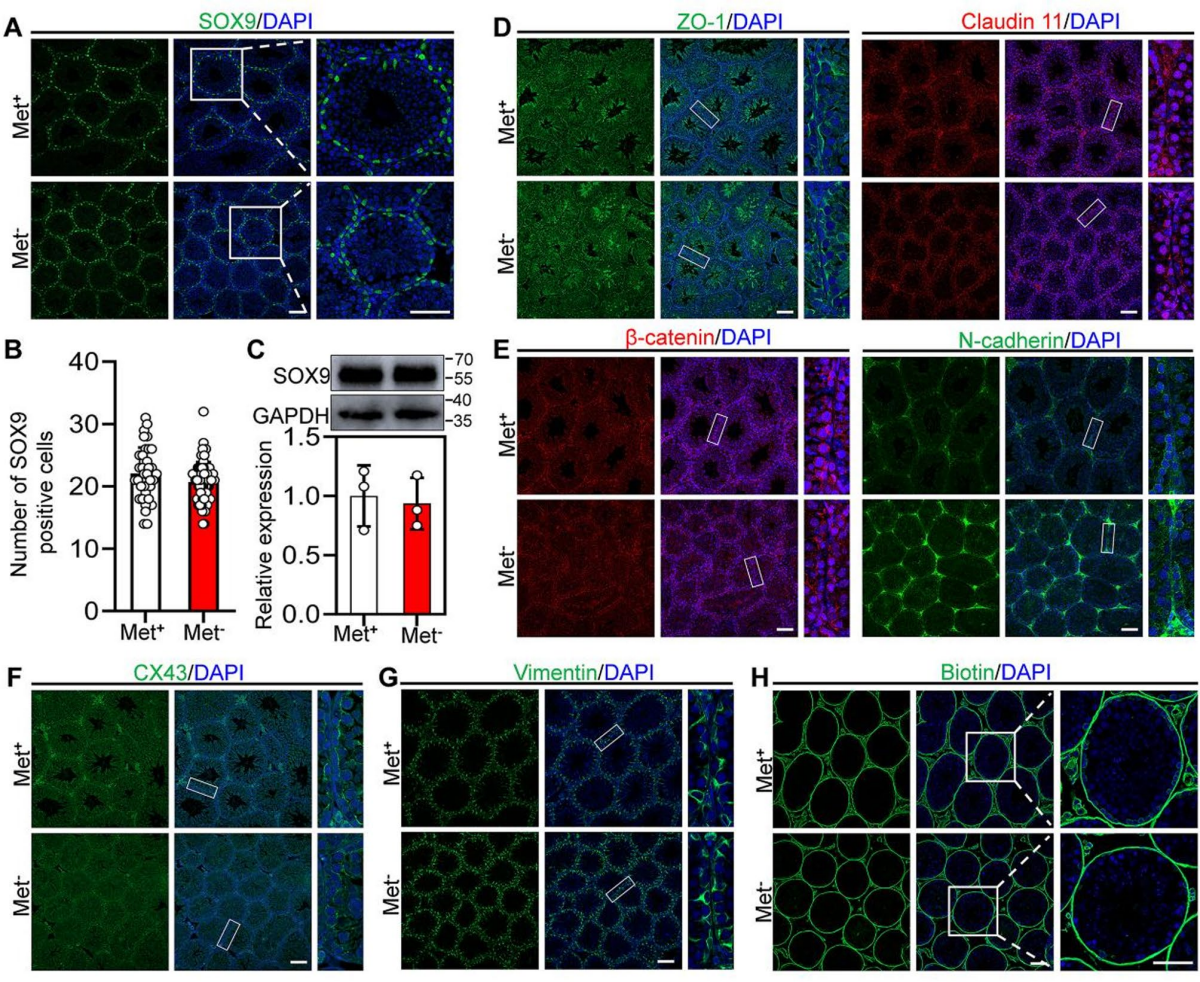
Methionine deprivation does not affect the integrity of the blood-testis barrier. **A** Immunofluorescence staining of SOX9 in testis from Met^+^ and Met^−^ mice. **B** Counting of SOX9 positive cells in testis from Met^+^ (38 seminiferous tubules from 3 mice) and Met^−^ (60 seminiferous tubules from 3 mice) mice. **C** Western blot analysis of SOX9 protein levels in testis from Met^+^ and Met^−^ mice, *n* = 3. **D**, **E**, **F**, **G** Immunofluorescence staining of ZO-1, Claudin 11, β-catenin, N-cadherin, CX43 and Vimentin in testis from Met^+^, MR and Met^−^ mice. (H) Integrity testing of the blood-testis barrier by intratesticular Biotin injection. Bars indicate the mean ± SD. A two-sided Student’s t‐test was used to determine *p*‐values. Scale bar = 100 μm

Sertoli cells play a critical role in the development and fate maintenance of Germ cells; thus, we systematically evaluated the function of Sertoli cells in mouse testes with methionine deprivation. Immunofluorescence staining and cell count showed that the SOX9-positive cells did not decrease in mouse testes with methionine deprivation compared with the control groups (Fig. 3A and B). Consistent with this finding, the protein levels of SOX9 in the testis showed no significant difference between the methionine-deprived and control group (Fig. 3C). Furthermore, the immunofluorescence staining of tight junction proteins (ZO1 and Claudin 11), basal ectoplasmic specializations protein complex (β-catenin and N-cadherin), gap junction protein (CX43), and intermediate filament cytoskeleton protein (Vimentin) showed similar expression levels and distribution pattern between the methionine deprivation and control group (Fig. 3D-G). The intratesticular injection of Biotin (a bio-tracer) was blocked outside of the seminiferous tubules in the methionine deprivation and the control group (Fig. 3H). These findings suggest that the integrity of the blood-testis barrier (BTB) was almost unaffected in mouse testes with methionine deprivation.

### Methionine deprivation caused a decrease in the number of mouse germ cells

**Fig. 4:**
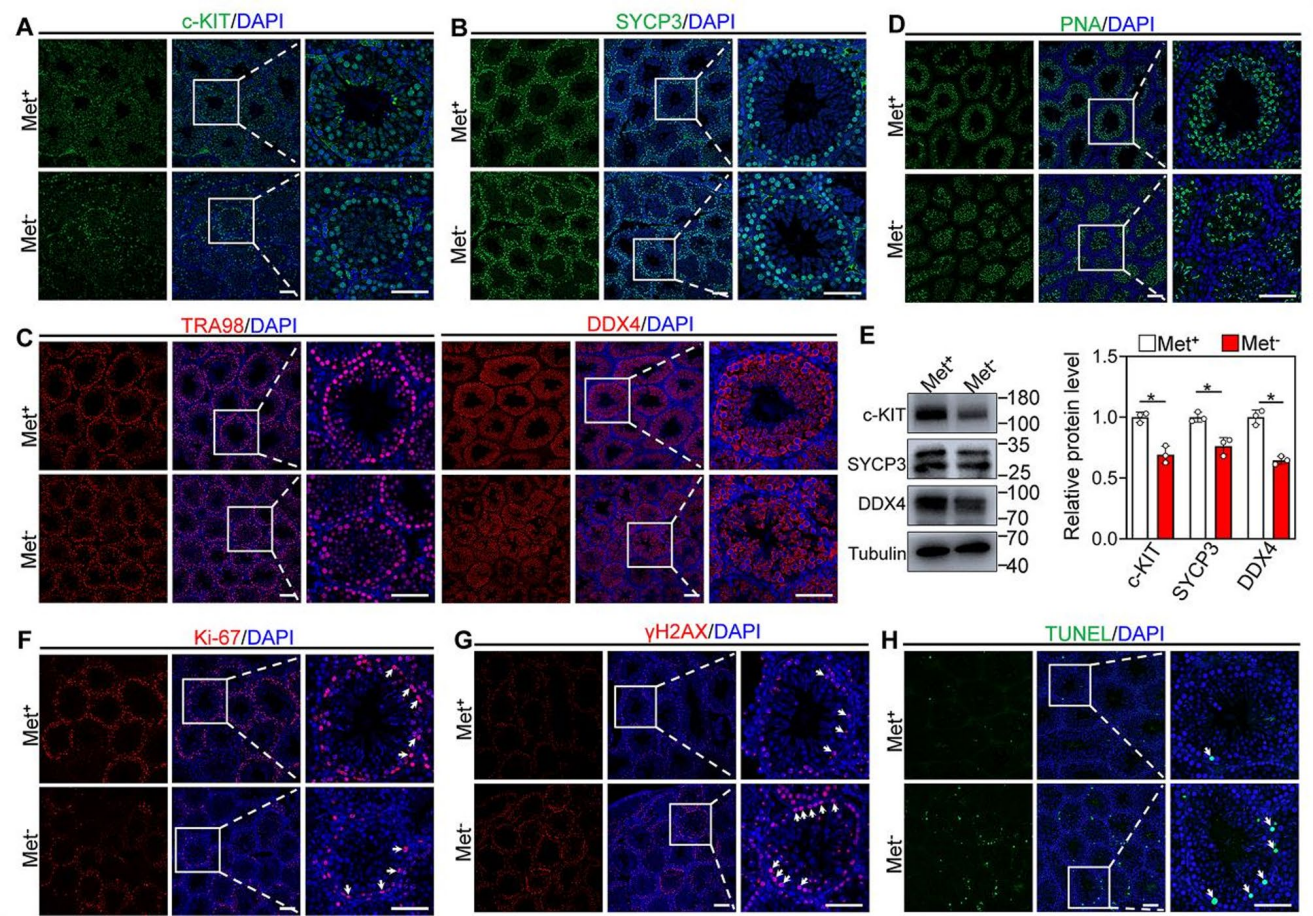
Methionine deprivation inhibits germ cell proliferation and leads to apoptosis. **A**, **B**, **C**, **D** Immunofluorescence staining of c-KIT, SYCP3, TRA98, DDX4 and PNA in testis from Met^+^ and Met^−^ mice. **E** Western blot analysis of c-KIT, SYCP3 and DDX4 protein levels in testis from Met^+^ and Met^−^ mice, *n* = 3. **F**, **G** Immunofluorescence staining of Ki-67 and γH2AX in testis from Met^+^ and Met^−^ mice. **H** Representative images of apoptosis analyzed by TUNEL staining in the testis from Met^+^ and Met^−^ mice. Bars indicate the mean ± SD. A two-sided Student’s t‐test was used to determine *p*‐values. (*p < 0.05). Scale bar = 100 μm

Considering the spermatogenesis impairment of mice in the methionine deprivation group, we performed immunofluorescence staining for spermatogenic cells at different stages. Immunofluorescence staining and counting showed that methionine deprivation led to a significant decrease in the number of c-KIT (a marker of spermatogonia)-positive cells per seminiferous tubule compared to the control group (Fig. 4A and S2A). Consistent with this, the number of SYCP3 (a marker of meiotic chromosome axis)-, TRA98 (a marker of germ cell)-, DDX4 (a marker of germ cell)- and PNA (a marker of acrosome)-positive cells in the testis with methionine deprivation showed a significant decrease relative to control groups (Fig. 4B-D and S2B-E). Western blot analysis also showed the protein levels of c-KIT, SYCP3 and DDX4 were downregulated in the testes with methionine deprivation (Fig. 4E). Further immunofluorescence staining and counting showed the Ki-67 (a marker of cell proliferation)-positive cells was significantly decreased in the testis with methionine deprivation compared with control groups, while the number of γH2AX (a marker of DNA double-strand break)-, TUNEL (a marker of apoptosis)- and c-PARP1 (a marker of apoptosis)-positive cells showed a significant increase (Fig. 4F-H and S2F-J). Furthermore, the co-staining results of c-KIT and Lamin B1 revealed that methionine deprivation caused a reduction in fluorescence intensity of Lamin B1 and loss of nuclear membrane in spermatogonia, further suggesting that these spermatogonia with methionine deprivation undergo apoptosis (Figure S2K). These findings indicate that methionine deprivation inhibits the proliferation of spermatogonia and promotes apoptosis in prepubertal mice, ultimately leading to defects in spermatogenesis.

### Methionine deprivation disturbs transcriptome homeostasis in GC-1 cells

**Fig. 5:**
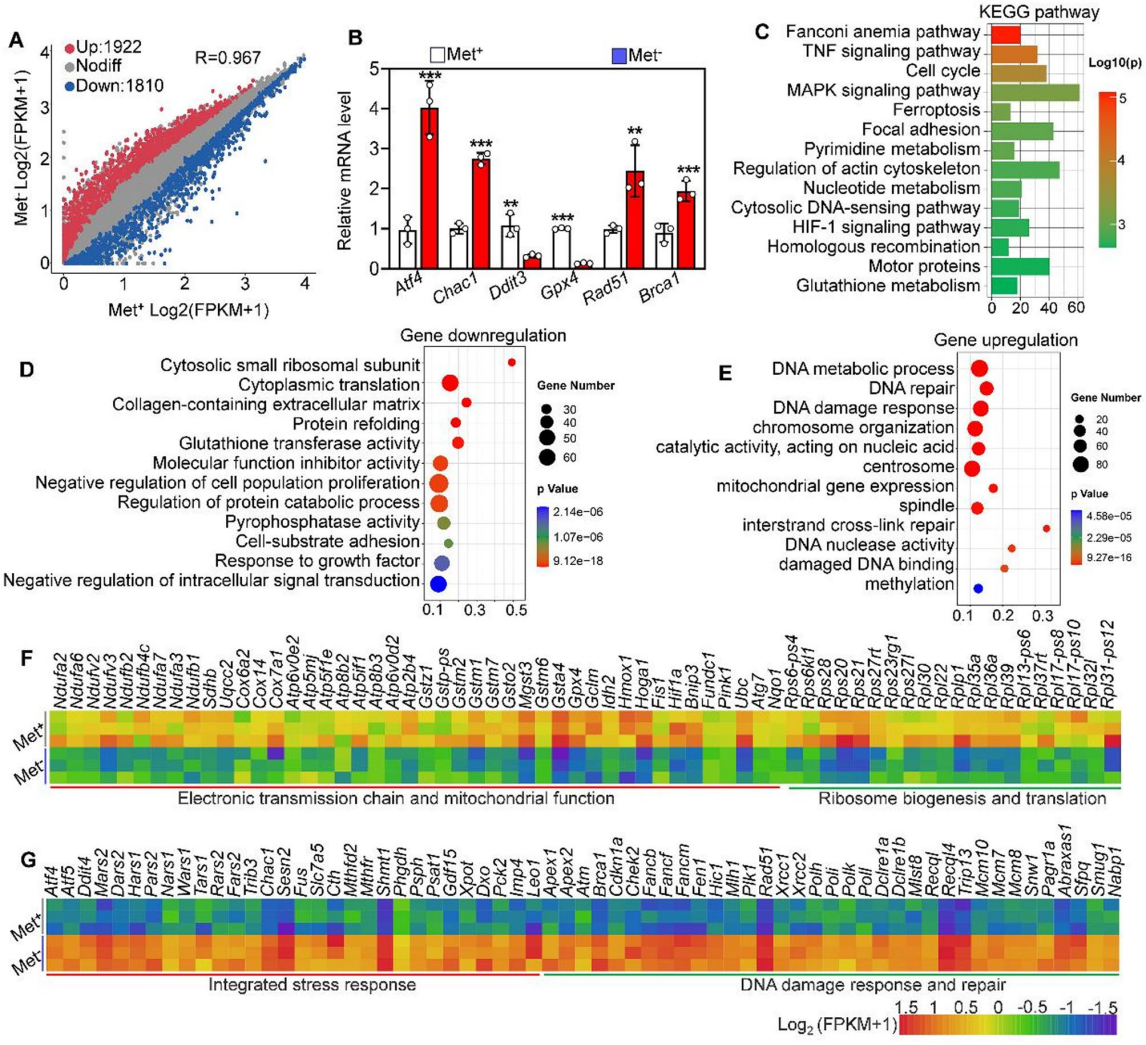
Methionine deprivation impairs the integrity of the transcriptome in GC-1 cells. **A** Scatter plot showing the transcripts with differential expression in Met^+^ and Met^−^ GC-1 cells. **B** qRT-PCR validating the differential transcripts identified by RNA‐seq, *n* = 3. **C** Bar chart illustrating the KEGG terms enriched by differential transcripts in Met^−^ GC-1 cells. **D**, **E** Bubble chart illustrating the GO terms enriched by downregulated and upregulated transcripts in Met^−^ GC-1 cells. **F**, **G** Heatmaps illustrating a group of downregulated and upregulated transcripts involved in the indicated biological processes in Met^+^ and Met^−^ GC-1 cells. Bars indicate the mean ± SD. A two‐sided Student’s t‐test was used to determine *p*‐values. (**p < 0.01 and ***p < 0.001)

To elucidate why germ cells in the testis are more sensitive to apoptosis under dietary methionine deprivation, we performed RNA-seq of GC-1 cells exposed to a methionine-free culture medium. The results showed that a total of 3732 transcripts (including 1922 upregulated and 1810 downregulated transcripts) were differentially expressed in the Met^−^ GC-1 cells compared with the control group (Fig. 5A). Some representative transcripts selected from RNA-seq data were verified by qRT-PCR (Fig. 5B). KEGG enrichment analysis showed that differentially expressed genes were involved in biological processes such as the Fanconi anemia pathway, cell cycle, ferroptosis, and glutathione metabolism (Fig. 5C). Further, we performed GO analysis of downregulated genes and upregulated genes, respectively. The results showed that the downregulated genes mainly participate in the electron transmission chain and mitochondrial function, cytoplasmic translation, protein refolding and glutathione transferase activity (Fig. 5D and F), while the upregulated genes were involved in integrated stress response, DNA damage response and repair, DNA metabolic process and methylation (Fig. 5E and G). Overall, the transcriptomic results reveal two critical insights. Firstly, methionine deprivation leads to mitochondrial dysfunction and triggers the integrated stress response (ISR) in GC-1 cells. Secondly, it also leads to an increase in DNA damage within these cells.

### Methionine deprivation causes GC-1 apoptosis via the DNA damage response pathway

**Fig. 6:**
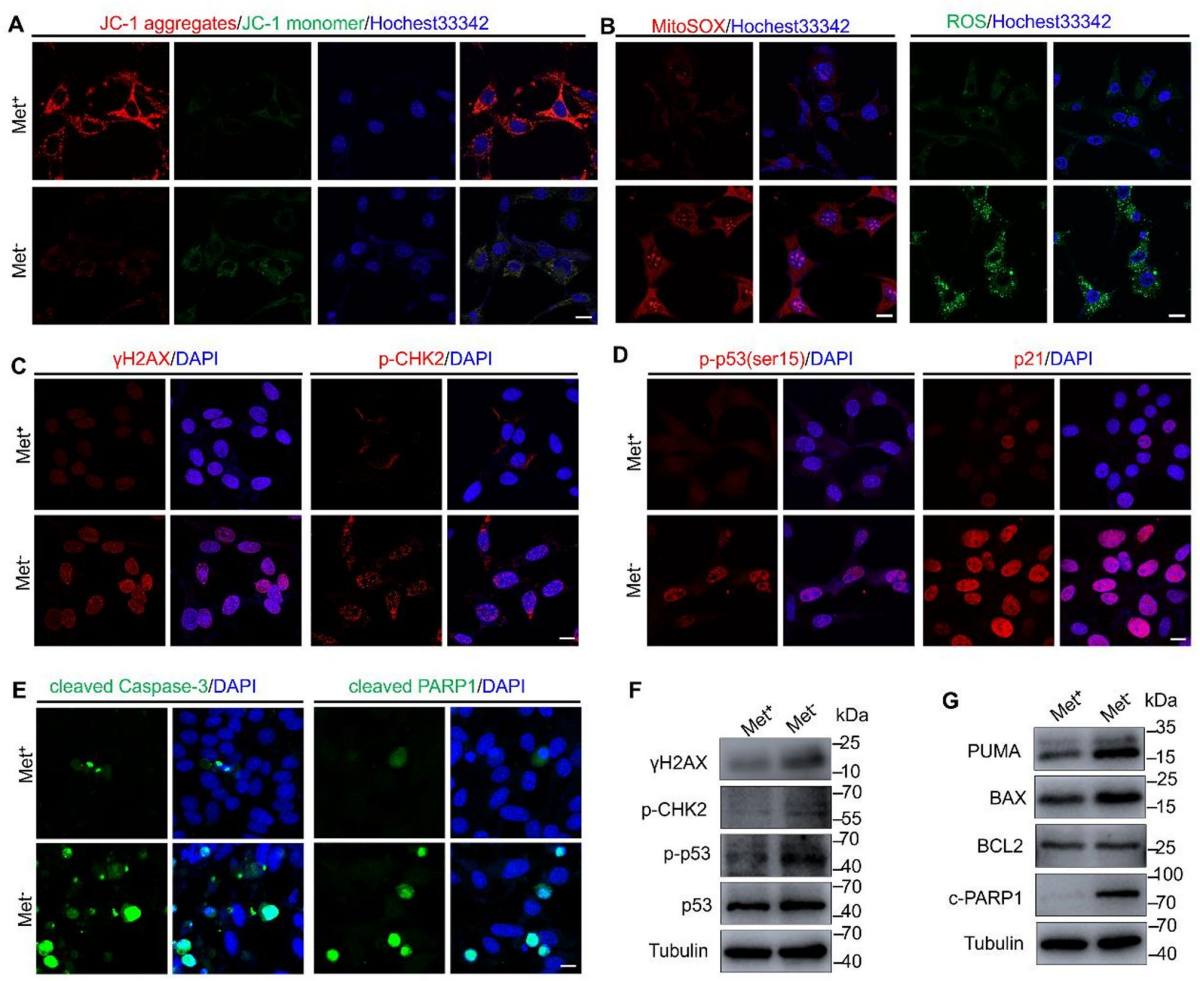
Methionine deprivation activates the oxidative stress and DNA damage response pathway in GC-1 cells. **A** Representative images of JC-1 staining in Met^+^ and Met^−^ GC-1 cells. **B** Representative images of ROS and MitoSOX staining in Met^+^ and Met^−^ GC-1 cells. **C**-**E** Immunofluorescence staining of γH2AX, p-CHK2, p-p53(ser15), p21, cleaved Caspase-3 and cleaved PARP1 in Met^+^ and Met^−^ GC-1 cells. (F, G) Western blot analysis of γH2AX, p-CHK2, p-p53, p53, PUMA, BAX, BCL2 and cleaved PARP1 protein levels in Met^+^ and Met^−^ GC-1 cells. Scale bar = 20 μm

Since genes related to mitochondrial function and DNA damage/repair were dysregulated in methionine-deficient GC-1 cells, it suggests that the GC-1 cells could undergo oxidative stress and DNA damage. JC-1 staining showed an obvious decrease in fluorescence intensity of aggregates but an obvious increase in monomers in Met^−^ GC-1 cells compared with the control group, representing depolarization of mitochondrial membrane potential (Fig. 6A). Meanwhile, the reactive oxygen species (ROS) and mitochondrial superoxide (MitoSOX) were excessively accumulated in Met^−^ GC-1 cells (Fig. 6B). Interestingly, although RNA-seq analysis revealed significant dysregulation of ferroptosis-related genes and pathways in Met^−^ GC-1 cells, no significant changes were observed in key ferroptosis markers—ferrous iron (Fe²⁺) levels and lipid peroxidation products (Figure S3). Immunofluorescence staining and Western blot analysis showed that DNA double-strand break marker γH2AX level and foci formation were increased in Met^−^ GC-1 cells relative to the control group (Fig. 6C and F). The increased foci of RAD51, a DNA repair protein in the homologous recombination pathway, further confirms that methionine deprivation induces DNA damage (Figure S4). This DNA damage signature was accompanied by the activation of effectors of the DNA damage response, such as phosphorylated CHK2 (p-CHK2), phosphorylated p53 (p-p53) and p21 (Fig. 6C, D and F). Consistent with these, the proapoptotic proteins, including PUMA, BAX, cleaved Caspase-3 and cleaved PARP1 in CG-1 cells were upregulated by methionine deprivation (Fig. 6E and G), while the anti‐apoptotic protein BCL2 was downregulated (Fig. 6G). These findings collectively reveal that methionine deprivation induces GC-1 cell apoptosis through DNA damage and subsequent DNA damage response pathways.

### NAC partially rescues mitochondrial function and GC-1 cell proliferation

**Fig. 7:**
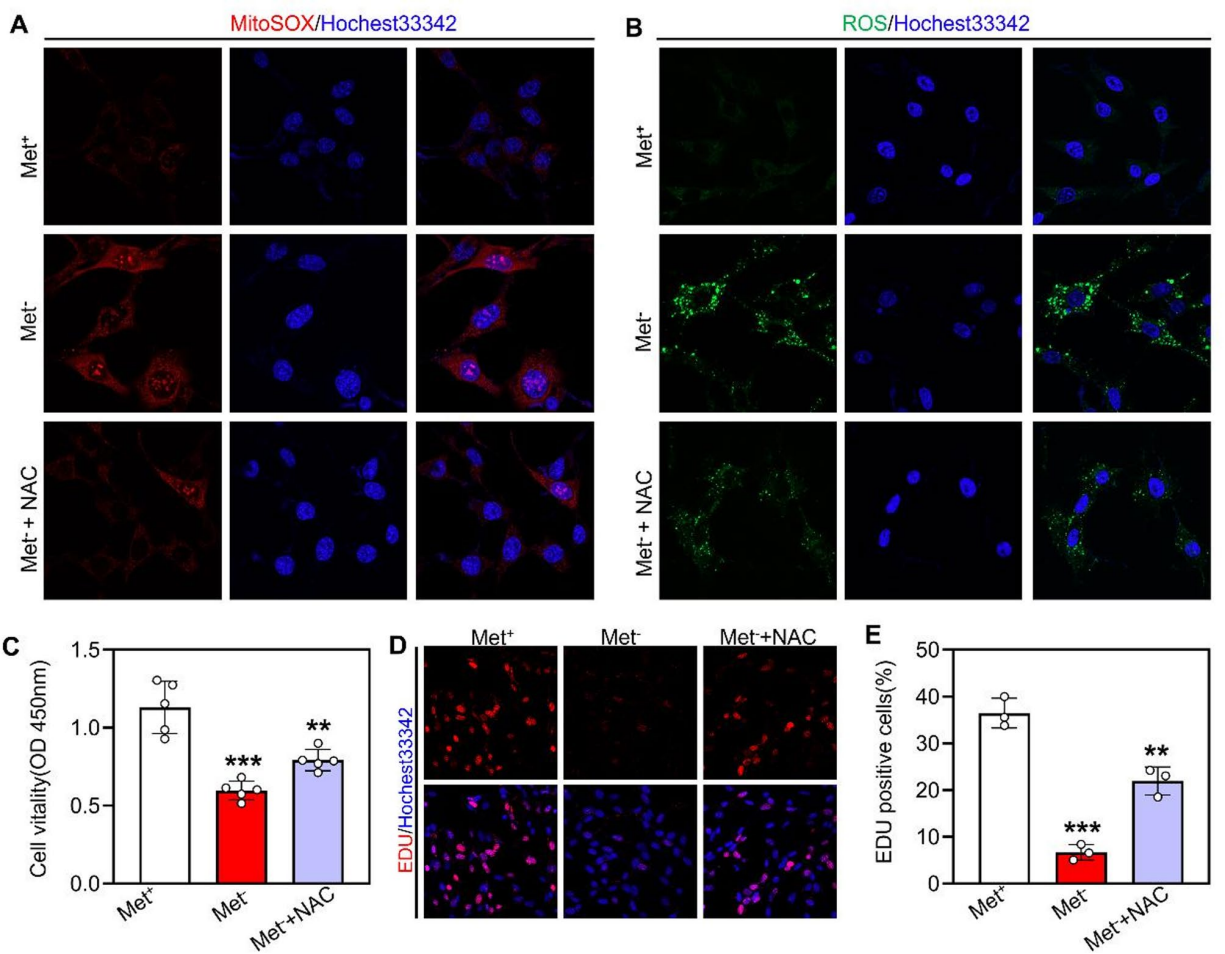
NAC alleviates methionine deprivation-induced proliferation inhibition and viability reduction in GC-1 cells. **A**,** B** Representative images of ROS and MitoSOX staining in Met^+^, Met^−^ and Met^−^ + NAC GC-1 cells. **C** CCK-8 assay for GC-1 cell viability, *n* = 5. **D** EdU incorporation assay for GC-1 cell proliferation. **E** Percentage of EDU positive GC-1 cells in Met^+^, Met^−^ and Met^−^ + NAC groups, *n* = 3. A one-way ANOVA followed by Tukey’s post hoc test was used to determine *p*‐values. (**p < 0.01 and ***p < 0.001). Scale bar = 20 μm

To further clarify methionine deprivation-induced oxidative stress and apoptosis, the GC-1 cells under methionine deprivation were treated using NAC (an effective antioxidant). The results showed that NAC treatment reduced the accumulation of both cellular ROS and MitoSOX in GC-1 cells (Fig. 7A and B), effectively reversing the oxidative stress triggered by methionine deprivation. Further, CCK8 detection results showed that the NAC treatment significantly increased GC-1 cell activity relative to the Met^−^ GC-1 cells (Fig. 7C). Furthermore, the EDU incorporation assay showed that the NAC treatment significantly enhanced GC-1 cell proliferation ability compared with Met^−^ GC-1 cells (Fig. 7D and E). These findings indicate that methionine deprivation-induced oxidative stress leads to defects in GC-1 proliferation and apoptosis.

## Discussion

Unbalanced diets are associated with developmental defects and various diseases. Our present study demonstrates that methionine intake restriction before puberty almost does not affect testicular development and spermatogenesis. However, the methionine-deficient mouse and GC-1 cells found that the methionine deprivation activated the oxidative stress signal and CHK2-p53/p21cascade, eventually leading to spermatogenesis impairment, cell cycle arrest and apoptosis of spermatogonia.

Seminiferous tubules are composed of Leydig cells, Sertoli cells, and germ cells at various stages of development [33, 34]. Developmental defects in any of these cells can lead to abnormal spermatogenesis. Amino acids are indispensable components for protein synthesis and are extremely important for the normal growth, differentiation, and maintenance of cellular functions [35]. Methionine, in particular, plays a direct role in regulating the assembly and availability of the protein translation initiation complex [36, 37]. Previous studies have found strong translational activity and weak anti-puromycin positive staining in spermatogonial stem cells and Leydig cells, respectively, within the testis through transient incorporation of puromycin [38]. These findings suggest that germ cells and Sertoli cells in the testis are sensitive to translation homeostasis and also reveal the potential causes of reduced proliferation and increased apoptosis in spermatogonia, as well as downregulated steroidogenic enzymes in Leydig cells caused by dietary methionine deprivation in our present study. Consistent with previous results on methionine-choline dietary deprivation, our study shows that short-term methionine deprivation around puberty does not significantly affect the integrity of the blood-testis barrier, possibly because the sensitivity of translational activity in Sertoli cells in the testis is relatively lower than in spermatogonia and Leydig cells [38]. Similarly, the knockout of the translation regulator RPL39L in mice led to a marked decrease in the number of spermatogonia but did not affect the number of Sertoli cells [38, 39]. Of course, whether long-term methionine deprivation will lead to impairment of Sertoli cell function and the integrity of the blood-testis barrier still deserves a follow-up study. Furthermore, our study found that dietary methionine restriction did not significantly affect early testicular development, which contrasts with recent reports that dietary methionine restriction extends the lifespan of *Drosophila melanogaster* but reduces their fertility [25]. The observed differences may be partly attributed to the shorter duration of dietary methionine restriction in our study. A comprehensive assessment of methionine restriction’s effects on testicular development might necessitate exposure to factors such as disease, aging, and various exogenous threats.

Integrated stress response (ISR) is an evolutionarily conserved self-protection mechanism activated when the body is exposed to threats such as stress, disease, and viral infection [40, 41]. Short-lived ISR is a pro‐survival response aiming at resolving stress and restoring homeostasis via translation reprogramming, while persistent or intense ISR can signal cell death induction [42, 43]. Consistent with previous studies in H1299 non-small cell lung cancer (NSCLC) cells, breast cancer cells and normal human fibroblasts [9, 44], our study also found that methionine deprivation induces ISR, characterized by upregulation of *Atf4*, *Chac1* and *Trib3*. As a pro-apoptotic molecule, CHAC1 induces programmed cell death characterized primarily by ferroptosis by degrading glutathione, affecting calcium signaling, and impairing mitochondrial respiratory function [45, 46]. However, our present study found no significant changes in the levels of lipid peroxides and ferrous ions, markers associated with ferroptosis, in GC-1 cells with methionine deprivation, suggesting that the activation of the ATF4-CHAC1 axis did not lead to ferroptosis in GC-1 cells. These results indicate that ferroptosis was suggested only by transcriptomic data and was not supported at the functional level. Exhaustion of glutathione impairs ROS homeostasis and leads to ROS accumulation, triggering subsequent cell apoptosis [47, 48]. The relationship between the degradation of glutathione caused by overexpression of CHAC1 and increased apoptosis has been established in yeast and macrophages, and the supplementation of glutathione could partially reverse apoptosis caused by overexpression of CHAC1 [49, 50]. Consistently, N-acetylcysteine (NAC, which not only directly neutralizes accumulated ROS but also provides precursors for endogenous glutathione synthesis) treatment increased the GC-1 cell proliferation by reducing ROS accumulation caused by methionine deprivation. All the above evidence suggests that methionine deprivation leads to loss of spermatogonia is caused by mitochondrial-mediated apoptosis. Nevertheless, future studies should employ NAC treatment in methionine-deprived mice to further elucidate the mechanisms underlying methionine deprivation-induced spermatogonial apoptosis, thereby enhancing our understanding of methionine’s role in early spermatogenesis.

During spermatogenesis, the germ cells are more susceptible to DNA damage due to active meiosis and mitosis, as well as threats from various external factors [51, 52]. Previous studies have found that the types of spermatogonia and round spermatids are more easily accumulated DNA damage compared with spermatogonial stem cells (PLZF-positive cells), which is confirmed by the mice exposed to radiation [53]. Consistently, our present study found that methionine deprivation resulted in significant accumulation of γH2AX in spermatogonia and GC-1 cells both in vivo and in vitro. Furthermore, methionine deprivation induced severe DNA damage in GC-1 cells also supported by the significantly enriched pathways related to DNA damage response and repair in the transcriptome. Notably, amino acid deprivation has been shown to increase ROS levels and decrease expression of the antioxidant factor NRF2 in human NSCLC cells and osteosarcoma cells [54, 55], which is consistent with our current finding that methionine deprivation elevates intracellular ROS levels in GC-1 cells. In the testis, ROS-induced DNA damage activates the ATR/ATM-CHK2-p53 axis, which subsequently induces cell cycle arrest through the activation of p21 or leads to cell apoptosis by activating apoptotic response factors such as PUMA [56–58].

In conclusion, the methionine deficiency mouse model and in vitro experiments collectively suggest that methionine deprivation-induced spermatogenesis defects and spermatogonia loss are primarily associated with the DNA damage response mediated by the ROS and CHK2-p53/p21 axis. Furthermore, our findings suggest that inhibition of ROS accumulation can partially alleviate methionine deprivation-induced impairment of spermatogonial proliferation in vitro. These results may provide valuable insights for maintaining dietary balance and potentially developing clinical interventions for related reproductive disorders.

## Supplementary Information

Below is the link to the electronic supplementary material.

Supplementary Material 1

## Data Availability

The datasets used and/or analyzed during the current study are available from the corresponding author on reasonable request. RNA-seq data have been deposited in the NCBI Gene Expression Omnibus (GEO) under accession number GSE308916. The original blot images have been submitted as Figure S5.
