# Supplementary material for: Methionine deficiency causes spermatogonial apoptosis via oxidative stress and DNA damage response pathway
Source: Biol Res. 2025 Nov 12;58:68. doi: 10.1186/s40659-025-00652-z (PMC12606962; doi:10.1186/s40659-025-00652-z)
Supplement: Supplementary file 1 — Supplementary Material 1 [file 40659_2025_652_MOESM1_ESM.docx]

**Supplementary Material**

Methionine deficiency causes spermatogonial apoptosis via oxidative stress and DNA damage response pathway

Weiyong Wang^1*^, Yong Ruan^1^, Ting Gong^1*^

**1** Key Laboratory of Animal Genetics, Breeding and Reproduction in the Plateau Mountainous Region, Ministry of Education, Guizhou University, Guiyang, Guizhou, 550025, China.

*Corresponding: **Weiyong Wang,** Key Laboratory of Animal Genetics, Breeding and Reproduction in the Plateau Mountainous Region, Ministry of Education, Guizhou University, Guiyang, Guizhou, 550025, China. E-mail: [wangwy08@outlook.com](mailto:wangwy08@outlook.com)

**Ting Gong,** Key Laboratory of Animal Genetics, Breeding and Reproduction in the Plateau Mountainous Region, Ministry of Education, Guizhou University, Guiyang, Guizhou, 550025, China. E-mail: tgong@gzu.edu.cn

This file includes:

Supplementary figures and figure legends

Supplementary tables

**
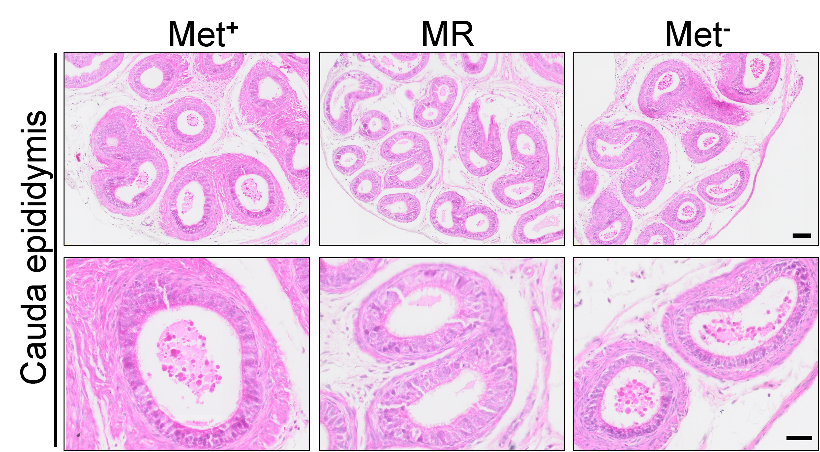
**

**Figure S1 HE staining of cauda epididymis from Met^+^, MR and Met^-^ mice. Bar = 100 µm.**

**
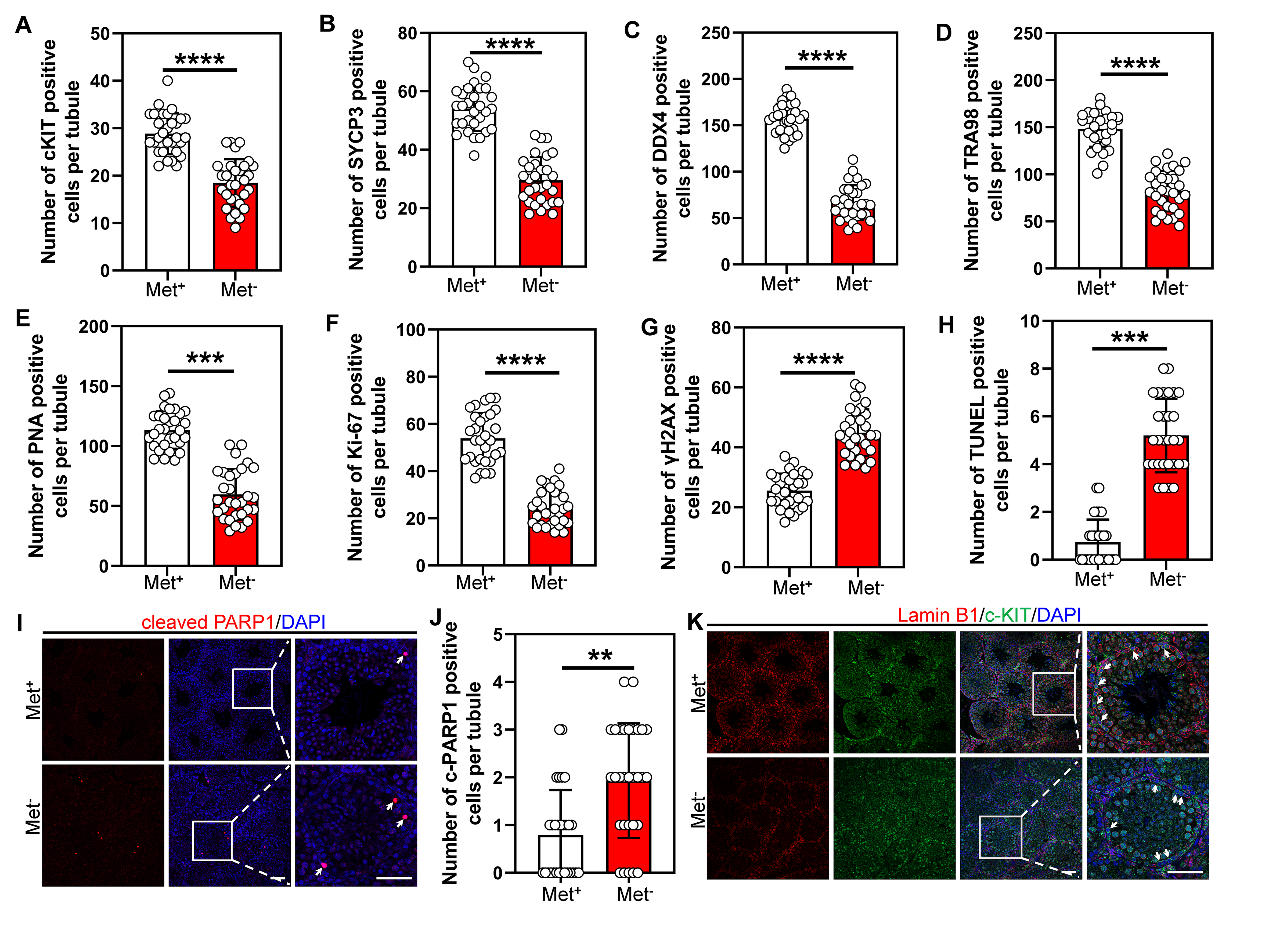
**

**Figure S2 Methionine deprivation inhibits germ cell proliferation and leads to apoptosis.** (A-H) Counting of c-KIT, SYCP3, DDX4, TRA98, PNA, Ki-67, γH2AX, and TUNEL positive cells in testis from Met^+^ and Met^-^ mice. (I) Immunofluorescence staining of cleaved PARP1 in testis from Met^+^ and Met^-^ mice. (J) Counting of cleaved PARP1-positive cells in the testis from Met^+^ and Met^-^ mice. (K) co-staining results of c-KIT and Lamin B1 cells in testis from Met^+^ and Met^-^ mice. Bars indicate the mean ± SD. A two‐sided Student's t‐test was used to determine *p*‐values. (**p* < 0.05, ***p* < 0.01, ****p* < 0.001, and *****p* < 0.0001). Scale bar = 100 µm.

**
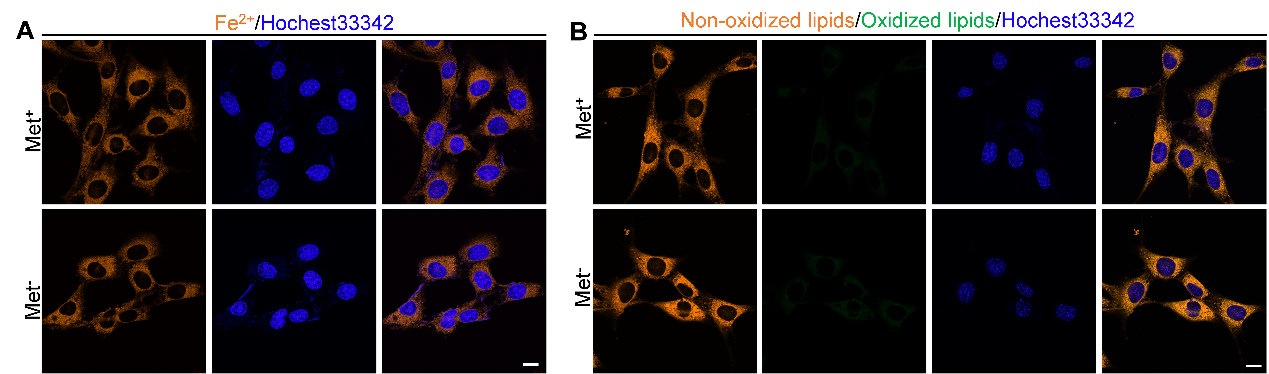
**

**Figure S3 Methionine deprivation did not cause ferroptosis in GC1 cells.** (A) Fe^2+^ staining in Met^+^ and Met^-^ GC-1 cells. (B) Lipid peroxide staining in Met^+^ and Met^-^ GC-1 cells. Scale bar = 20 µm.

**
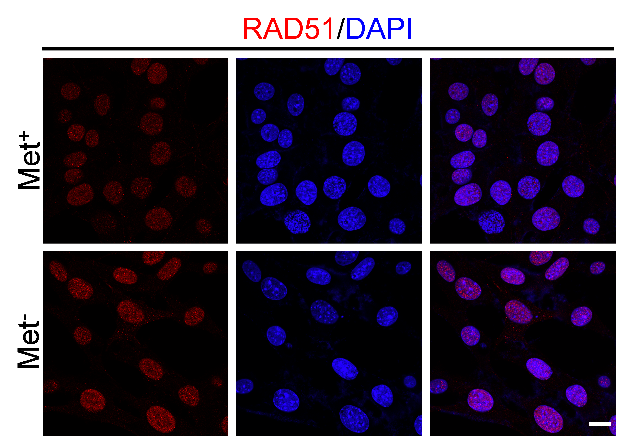
**

**Figure S4. Immunofluorescence staining of RAD51 in Met+ and Met- GC-1 cells.** Scale bar = 20 µm.

**
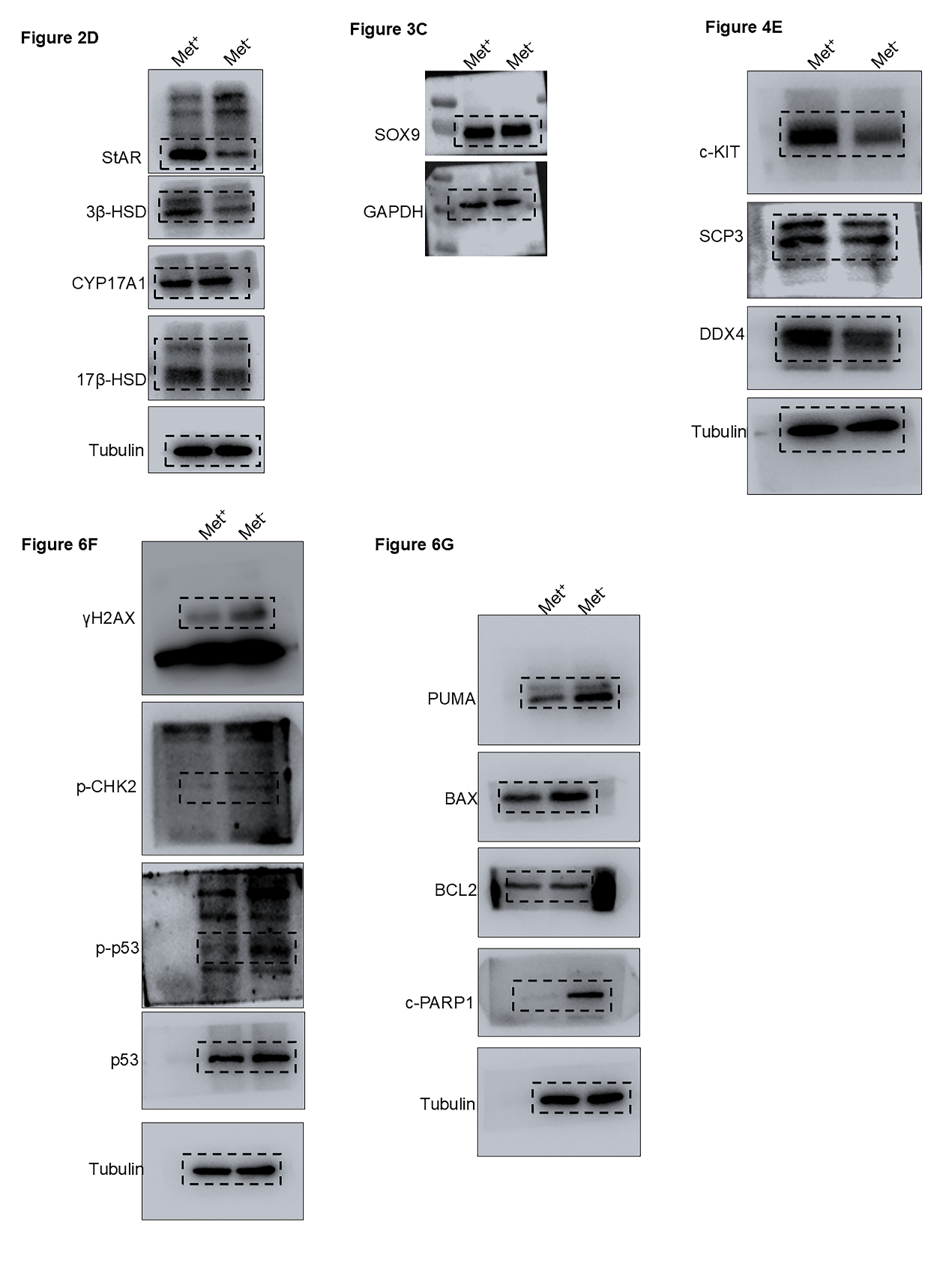
**

**Figure S5. Original blots of Figure 2D, 3C, 4E, 6F and 6G.**

**Table S1. Primers for qRT-PCR**

| Genes | Forward primers (5’-3’) | Reverse primers (5’-3’) | |
| --- | --- | --- | --- |
| *Atf4* | CCTATAAAGGCTTGCGGCCA | CACTGCTGCTGGATTTCGTG | |
| *Chac1* | CTGTGGATTTTCGGGTACGG | CCCCTATGGAAGGTGTCTCC | |
| *Ddit3* | CTGGAAGCCTGGTATGAGGAT | CAGGGTCAAGAGTAGTGAAGGT | |
| *Gpx4* | GCCTGGATAAGTACAGGGGTT | CATGCAGATCGACTAGCTGAG | |
| *Rad51* | AAGTTTTGGTCCACAGCCTATTT | CGGTGCATAAGCAACAGCC |  |
| *Brca1* | AAGTGCCAGTGTCAAGGAGA | ACAGGGAGCAAAAGGGAAGA |  |
| *Actb* | GGCTGTATTCCCCTCCATCG | CCAGTTGGTAACAATGCCATGT |  |

**Table S2. List of primary antibodies used in immune detection**

| Antibody | Catalog Code | | | Source | | Host | | | Dilution | | |
| --- | --- | --- | --- | --- | --- | --- | --- | --- | --- | --- | --- |
|  |  |  |  |  |  |  |  |  | IF | | WB |
| PNA | | | L21409 | | Thermo Fisher | |  | 1:200 | |  | |
| StAR | | | A1035 | | Abclonal | | Rabbit | 1:100 | | 1:1000 | |
| 3β-HSD | | | sc-515120 | | Santa Cruz | | Mouse | 1:100 | | 1:500 | |
| 17β-HSD | | | PTM-6672 | | PTM Bio | | Rabbit | 1:200 | | 1:1500 | |
| CYP17A1 | | | PTM-7192 | | PTM Bio | | Rabbit |  | | 1:1000 | |
| SOX9 | | | ab185966 | | Abcam | | Rabbit | 1:100 | | 1:500 | |
| ZO-1 | | | 40-2200 | | Invitrogen | | Rabbit | 1:100 | |  | |
| Claudin 11 | | | 36-4500 | | Invitrogen | | Rabbit | 1:200 | |  | |
| β-catenin | | | 712700 | | Invitrogen | | Rabbit | 1:50 | |  | |
| N-cadherin | | | sc-59987 | | Santa Cruz | | Mouse | 1:100 | |  | |
| CX43 | | | 3512 | | CST | | Rabbit | 1:200 | |  | |
| Vimentin | | | 5741s | | CST | | Rabbit | 1:200 | |  | |
| GAPDH | | | 5174 | | CST | | Rabbit |  | | 1:1000 | |
| c-KIT | | | AF1356-SP | | R&D Systems | | Goat | 1:200 | | 1:1000 | |
| SYCP3 | | | [sc-74569](https://www.scbt.com/zh/p/scp-3-antibody-d-1?requestFrom=search) | | Santa Cruz | | Mouse | 1:50 | | 1:1000 | |
| TRA98 | | | ab82527 | | Abcam | | Rabbit | 1:100 | |  | |
| DDX4 | | | ab27591 | | Abcam | | Rabbit | 1:200 | | 1:1000 | |
| Ki-67 | | | 9129s | | CST | | Rabbit | 1:200 | |  | |
| γH2AX | | | ab22551 | | Abcam | | Rabbit |  | | 1:1000 | |
| γH2AX | | | ab206900 | | Abcam | |  | 1:300 | |  | |
| α-tubulin | | | 66031-1-IG | | Proteintech | | Rabbit |  | | 1:2000 | |
| c-PARP1 | | | 5625 | | CST | | Rabbit | 1:200 | | 1:1000 | |
| Lamin B1 | | | HY-80205 | | MCE | | Rabbit | 1:200 | |  | |
| Phospho-p53-ser15 | | | 9284T | | CST | | Rabbit | 1:200 | | 1:1000 | |
| PUMA | | | 98672 | | CST | | Rabbit |  | | 1:500 | |
| Phospho-CHK2 | | | HY-80799 | | MCE | | Rabbit | 1:200 | | 1:500 | |
| RAD51 | | | ab133534 | | Abcam | | Rabbit | 1:200 | |  | |
| p53 | | | sc-126 | | Santa Cruz | | Mouse |  | | 1:500 | |
| p21 | | | ab188224 | | Abcam | | Rabbit | 1:100 | |  | |
| cleaved Caspase-3 | | 9664 | | | CST | | Rabbit | 1:100 | |  | |
| BAX | | 50599-2-Ig | | | Proteintech | | Rabbit |  | | 1:1000 | |
| BCL2 | | 26593-1-AP | | | Proteintech | | Rabbit |  | | 1:1000 | |

IF: Immunofluorescence; WB: Western blotting

**Table S3. Percentage change in body weight of mice in Met^+^, MR and Met^-^ groups**

| Age | Weight (g)  Met^+^ group | Percentage change (%) | Weight (g)  MR group | Percentage change (%) | Weight (g)  Met^-^ group | Percentage change (%) |
| --- | --- | --- | --- | --- | --- | --- |
| PD25 | 9.38 ± 1.25 | -- | 9.16 ± 0.92 | -- | 9.52 ± 1.09 | -- |
| PD28 | 10.71 ± 1.28 | 14.18 | 10.62 ± 1.33 | 15.94 | 8.87 ± 0.79 | -6.83 |
| PD31 | 13.02 ± 1.17 | 21.57 | 12.94 ± 1.24 | 21.85 | 8.31 ± 0.43 | -6.31 |
| PD34 | 14.63 ± 1.22 | 12.37 | 15.37 ± 1.51 | 18.78 | 8.37 ± 0.54 | 0.72 |
| PD37 | 16.24 ± 1.09 | 11.01 | 16.39 ± 1.78 | 6.64 | 8.24 ± 0.6 | -1.55 |
| PD40 | 17.11 ± 1.52 | 5.37 | 17.7 ± 1.77 | 7.99 | 8.31 ± 0.52 | 0.84 |
